# Experimental Infection of Sand Flies by Massilia Virus and Viral Transmission by Co-Feeding on Sugar Meal

**DOI:** 10.3390/v11040332

**Published:** 2019-04-09

**Authors:** Magdalena Jancarova, Laurence Bichaud, Jana Hlavacova, Stephane Priet, Nazli Ayhan, Tatiana Spitzova, Petr Volf, Remi N. Charrel

**Affiliations:** 1 Department of Parasitology, Faculty of Science, Charles University, 128 44 Prague 2, Czech Republic; magda.jancarova@seznam.cz (M.J.); laurencebichaud@gmail.com (L.B.); janehlavac@seznam.cz (J.H.); tatiana.spitzova@gmail.com (T.S.); volf@cesnet.cz (P.V.); 2 Unité des Virus Emergents (UVE: Aix Marseille Univ, IRD 190, INSERM 1207, IHU Méditerranée Infection), 13005 Marseille, France; stephpriet@gmail.com (S.P.); nazliayhann@gmail.com (N.A.)

**Keywords:** *Phlebovirus*, virus transmission, *Phlebotomus*, *Lutzomyia*, *Sergentomyia*, Phenuiviridae, Toscana virus, sand fly

## Abstract

**Background:** Massilia virus (MASV) is a phlebovirus isolated from *Phlebotomus perniciosus* in various regions of southwestern Europe. It is closely related to human pathogens such as Toscana virus and sandfly fever Naples virus. The natural cycle of phleboviruses is poorly understood. Indeed, experimental studies demonstrate that transovarial and sexual transmission are not efficient enough for the maintenance of the virus in nature and to date there is no convincing evidence that a species of vertebrates is the reservoir of the virus. Here, we studied various transmission routes of MASV taking advantage of experimental colonies representing different species of sand flies. **Methodology/Principal findings:** In *P. perniciosus*, four sources of infection were compared: (i) Virus-seeded larval food to the first instar larvae (L1), or (ii) to the fourth instar larvae (L4), (iii) virus-seeded blood meal to adult females, and (iv) virus-seeded sugar meal to adults of both sexes. From 875 adults emerged from infected L1 and L4, only three were positive. In females infected by bloodmeal the infection rate was high before defecation, then it decreased drastically; MASV RNA was detected in only 5 out of 27 post-defecation. Surprisingly, the most efficient route of infection was observed after intake of virus-seeded sugar meal: 72% of females (79/110) and 52% of males (51/99) were found to be MASV RNA-positive. In addition, MASV-infected sandflies regurgitated virus particules into the sugar drop and MASV RNA was detectable in this drop for at least 24 h after regurgitation. MASV RNA was detected in about one third of the *P. perniciosus* exposed to this sugar drop contaminated by regurgitation. Sugar meal infection was also tested with six other species of sand flies. In males, there were no significant differences in infection rates when compared to *P. perniciosus*. In females, most species tested showed high infection rate at the beginning but then significant gradual decrease in infection rate during the experiment. **Conclusions/Significance**: We present the first description of arboviral infection of a dipteran vector using sugar meal. In all seven sand fly species tested, MASV was detected for two weeks post-infection. Our results showed that MASV can be transmitted between *P. perniciosus* either through co-feeding or via an infected sugar source such as plant sap. These newly described routes of horizontal transmission may play an important role in the circulation of phleboviruses in nature.

## 1. Introduction

Phlebotomine sand flies (Diptera: Phlebotominae) occur in a wide variety of habitats, from deserts to rainforests. Both adult sexes feed on a natural sugar sources, such as sap of plants or honeydew, but females feed also on blood of various vertebrates to obtain proteins required for egg development. Two genera, *Phlebotomus* in the Old World and *Lutzomyia* in the New World, frequently bite humans. Consequently, members of these two sand fly genera are known vectors of human or animal pathogens, namely *Leishmania* parasites, bacteria (e.g., *Bartonella bacilliformis*), and viruses belonging to genera *Phlebovirus*, *Vesiculovirus,* and *Orbivirus* [1,2,3].

According to the International Committee on Taxonomy of Viruses (ICTV) [4], the genus *Phlebovirus* (Phenuiviridae family within the order Bunyavirales) includes nine viral phleboviral species (Bujaru, Candiru, Chilibre, Frijoles, Punta Toro, Rift Valley fever, Salehabad, Sandfly fever Naples, and Uukuniemi) and 33 tentative species. Most of them are transmitted by phlebotomine sand flies, however, Uukuniemi virus and Rift Valley virus are vectored preferably by ticks and mosquitoes, respectively [3,5,6].

Some phleboviruses cause human diseases with variety of clinical syndromes, from mild febrile to neuroinvasive disease. Among those circulating in Mediterranean area, Toscana virus (TOSV) is the most important for medical reasons. TOSV infection can present with a large variety of symptoms from very mild febrile illness to severe neuroinvasive infections, such as meningitis, encephalitis, and peripheral neurological manifestations [7,8]. Studies performed during the last decade have shown that TOSV is present in countries bordering the Mediterranean, with at least 250 million people at risk of infection [8,9]. Apart from *Phlebotomus perniciosus* and *Phlebotomus perfiliewi*, there is increasing evidence demonstrating that there are other potential vectors of TOSV, such as *Sergentomyia minuta* [10], *Phlebotomus tobbi* [11], *Phlebotomus neglectus* [12], *Phlebotomus longicuspis,* and *Phlebotomus sergenti* [9,13]. 

Massilia virus (MASV), discovered and characterized in 2005, belongs to the *Sandfly fever Naples phlebovirus* species, as TOSV; accordingly, MASV and TOSV are highly similar in terms of physical and antigenic properties. MASV was isolated from *P. perniciosus* in several regions where TOSV is also present, indicating that both viruses can co-circulate and are transmitted by the same vectors [14].

The circulation of sand fly-borne phleboviruses in nature is poorly understood. There is no convincing evidence supporting the existence of human or animal vertebrate reservoirs; vertebrates are considered as dead-end hosts without significant role in life cycle of the virus, while sand flies are suggested as primary reservoirs by some authors [3,15]. Experimental studies demonstrated transovarial [16,17,18] and sexual [17] transmission of TOSV between sand flies, however, these ways of transmission are not efficient enough for the maintenance of these viruses in nature [16,17] and non-viraemic transmission known in ticks [19], mosquitoes [20], and black flies [21] was not demonstrated yet in sand flies. Therefore, some other alternative routes or different combinations of transmission could be expected. 

Here, we used MASV as a safe laboratory model (i) for gaining understanding in the mechanisms of phlebovirus transmission within *Phlebotomus perniciosus* sand flies and (ii) to compare results obtained with *P. perniciosus* with sand flies belonging to other *Phlebotomus* species and to other genera such as *Lutzomyia* and *Sergentomyia*.

## 2. Material and Methods

### 2.1. Sand Fly Colonies

Seven sand fly colonies were reared at Laboratory of Vector Biology, Charles University in Prague for many generations. The colony of the New World sand fly *Lutzomyia longipalpis* was established from females caught in Brazil. Six Old World colonies were represented by *P. perniciosus* (originated from Spain), *Phlebotomus papatasi* and *P. sergenti* (both from Turkey), *Phlebotomus orientalis* and *Sergentomyia schwetzi* (both from Ethiopia), and *Phlebotomus argentipes* (from India). Unless otherwise specified, sand flies were maintained under standard conditions as described previously [22]. Before the experiments, each colony was tested for the presence of MASV and all were negative.

### 2.2. Virus Strain

All experiments were performed using Massilia virus strain UVE/MASV/2009/FR/M43(Ref-SKU#001V-02369 https://www.european-virus-archive.com/virus/massilia-virus-strain-uvemasv2009frm43). Each vial contained 0.2 mL of lyophilized virus containing 3.32 × 10^6^ TCID_50_, which is the standard dose used when l-MASV is mentioned in the next paragraphs.

### 2.3. Different Infection Routes for MASV Infection in Sand Flies

*Phlebotomus perniciosus* was used to study and compare the effectiveness of infection in 4 types of sand fly developmental stages through different pathways: (i) In larval food to the first instar larvae (L1), (ii) in larval food to the fourth (last) instar larvae (L4), (iii) by blood meal to adult females, and (iv) by sugar meal to adults of both sexes. For infections of L1 and L4, l-MASV was diluted in 500 µL of sterile distilled water (6.64 × 10^6^ TCID_50_/mL) and mixed with 500 µL of larval food consisting of composted rabbit chow and rabbit feces as described [22]. 

**L1 larvae** kept in breeding pots were given this mixture on day 0 and day 2 of the experiment (D0 and D2). Then, 20 larvae were collected at various intervals (D2, D5, D12, and D19) and individually stored in 70% ethanol. From the same breeding pot, 20 pupae were collected on D26 and freshly emerged adults on twelve different intervals (D33, D35, D37, D38, D40, D42, D45, D47, D49, D52, D54, D56). All specimens were again stored individually in 70% ethanol. 

Actively feeding **L4 larvae** were infected by larval food by a single infected food (D0) as described above. Ten larvae and ten pupae were collected on days D2 and D5, respectively. All adults emerging from the same container were collected at seven different intervals (D12, D14, D16, D19, D21, D23, D26) and individually stored in 70% ethanol. 

For **blood meal infections** of adult *P. perniciosus* females the l-MASV was diluted in 200 µL of sterile distilled water, mixed with 1.8 mL of heat-inactivated rabbit blood (1.66 x 10^6^ TCID_50_/mL) and fed to approximately 150 females through a chick-skin membrane using a glass feeder (similar to *Leishmania* experimental infections described [22]). Fully engorged females were separated into a new cage and kept at 26 °C. Females collected on days D0, D5, D7, D9 (10, 10, 10, and 7 females, respectively) were stored individually in 70% ethanol. 

For **sugar meal infections**, groups of freshly emerged adults, 200 males and 200 females, were separated into two cages and offered a mixture of 500 µL of sugar with l-MASV (6.64 × 10^6^ TCID_50_/mL). The sugar solution was the same as used for maintenance of sand fly colonies (50% brown sugar in sterile distilled water prepared as described [22]). The fresh mixtures of sugar with l-MASV were soaked into a small piece of cotton wool placed on a small glass Petri dish and offered to sand flies twice, on D0 and D2. About ten males and ten females were then collected at 10 time intervals (D5, D7, D9, D12, D14, D16, D19, D21, D23, and D26) and stored as described above. 

### 2.4. MASV Detection in Experimentally Infected Sand Flies

All samples of various developmental stages (larvae, pupae, and adults) were processed at the Unité des Virus Emergents in Marseille. Before processing, L1 and L4 were washed in physiological solution to remove larval feces and particles of infectious food. Each sample was homogenized individually in 600 μL of Eagle minimal essential medium (EMEM) supplemented with 7% fetal bovine serum, 1% penicillin-streptomycin, and 1% L-glutamine (200 mM) using a Mixer Mill MM300 (Qiagen, Courtaboeuf, France) in the presence of a 3-mm tungsten bead. The resulting homogenate was centrifugated at 5000 g for 5 min to separate supernatant. 200 μL of supernatant was processed further and rest was stored at −80 °C. Viral nucleic acid was extracted by the Virus Extraction minikit (Qiagen) by BioRobot EZ1-XL Advanced (Qiagen) and eluted into 90 μL. Five microliters of this solution was used for real-time RT-PCR performed by SuperScript® III Platinum® One-Step qRT-PCR Kit w/ROX (Invitrogen, Villebon sur Yvette, France) according to manufacturer’s protocol on a CFX96 real-time system (Bio-Rad, Marnes la Coquette, France): (i) 48 °C for 30 min, (ii) 95 °C for 2 min, (iii) 95 °C for 30s, (iv) 60 °C for 1 min; steps (iii) and (iv) were repeated 45x. Primers and probes designed for the nucleoprotein gene specific for MASV were described previously [14].

### 2.5. Co-Feeding Experiments (CoF)

We investigated whether infected sand flies spit the virus during sugar feeding (CoF1 experiments) or not and then if virus particles released in the sugar meal are capable of infecting naïve sand flies (CoF2). For CoF2, we used the term “co-feeding on sugar” to distinguish from co-feeding on vertebretes as non-viremic transmission described previously [19,20].

In CoF1 experiments, 100 females and 100 males were exposed to l-MASV infected sugar meal on days D0 and D2 as described above. From D4, groups of infected sand flies were subsequently offered a non-infected sugar meal. A small drop of sugar on parafilm in a glass Petri dish was placed into the cage with 100 infected sand flies for 24 h, after which the sugar drop was collected and used for MASV RNA detection using qRTPCR. This was repeated 3 times. 

**Figure 1:**
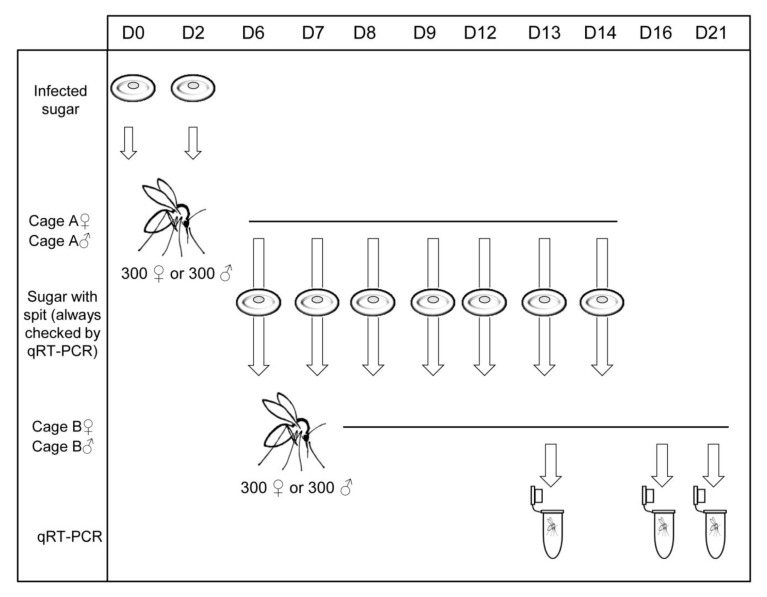
Schema of the co-feeding (CoF2) experiment.

In the second experiment (CoF2), groups of 300 females and 300 males were separated into two cages (cage A♀ and A♂) and provided l-MASV-infected sugar meal at D0 and D2. From D6 to D14, they were offered the aforementioned non-infected sugar meal. The next day we removed the sugar meal, took 140 µL for RT-PCR, and transferred the rest to another cages (B♀ and B♂) with non-infected females and males to test if spitted MASV is infectious for other sand flies. Sand flies from cages B♀ and B♂ were removed at D13, D16, and D21, stored in 70% ethanol individually, and examined for presence of MASV as described above. The scheme of this experiment is depicted in Figure 1.

### 2.6. Susceptibility of Various Sand Fly Species to MASV

Seven species belonging to three sand fly genera, *Phlebotomus*, *Lutzomyia*, and *Sergentomyia*, were used: *P. perniciosus* (positive control), *P. orientalis*, *P. papatasi*, *P. sergenti*, *P. argentipes*, *S. schwetzi*, and *L. longipalpis*. For each species, 250 females and 250 males were offered a l-MASV infected sugar meal at D0 and D2. Then, up to 10 males and 10 females were collected at intervals up to eight times (D2, D4, D7, D9, D11, D14, D16, D21) and stored individually in 70% ethanol. The experiment was repeated two times with different sand flies.

### 2.7. Tissue Specificity of MASV

Females of three species belonging to three sand fly genera were chosen: *P. perniciosus*, *S. schwetzi*, and *L. longipalpis*. 200 females of each species were infected by sugar meal at D0 and D2. At D4, D7, and D10, 10 females were dissected to obtain the salivary glands, the midgut, and the rest of body separately for each sand fly. During dissection, the salivary glands and the midgut were washed in a drop of sterile physiological solution to prevent contamination by another tissue. All samples were homogenized in 20 µL of EMEM by automatic homogenizer, then 120 µL of EMEM was added. Viral nucleic acid was extracted from the entire sample (140 µL) and RT-PCR for detection of MASV RNA was performed as described above.

### 2.8. Statistical Analysis

Statistical analyses were carried out using R software (http://cran.r-project.org/). Association between infection and the infection route (blood, sugar) was analysed using Chi^2^ test. The development of infection in *P. perniciosus* males and females was compared by fitting Generalised linear models (GLM) with quiasi-binomial distribution with interaction terms included. The relationship between virion loads and time and infection rate and time among the tested colonies were tested by fitting GLM with quasi-poisson distribution and with quasi-binomial distribution, respectively. Used models included interaction terms to test differences between the colonies. The relationship between virion loads in different tissues (salivary glands, gut, and the rest of the body) and time among the tested colonies were analyzed by fitting GLMs with quasi-poisson distribution with interaction terms included in order to test differences between colonies. A p-value of <0.05 was considered to indicate statistical significance. The results were graphically presented using the “ggplot2“ package in R software.

## 3. Results

### 3.1. Infection of P. perniciosus L1 Using Larval Food

Larvae of the first instar (L1) were infected by virus suspension mixed with larval food. Out of 78 infected L1 collected from D2 to D19, MASV was detected only in six larvae collected at D5. Forty larvae collected from D12 to D19 were negative. Low positivity (5%) was detected in pupae; one out of 20 collected on D26 was found positive for MASV. Even lower positivity was found in adults. Among the 796 adults emerged from infected L1 between D33 to D56 (399 females and 397 males together), only one male (D35) was positive for MASV. Data are summarized in Table S1.

### 3.2. Infections of P. perniciosus L4 by Larval Food

Larvae of the fourth instar (L4) were infected by virus suspension mixed with larval food. All 30 L4 of *P. perniciosus* collected at D0 (*n* = 20) and D2 (*n* = 10) were negative. From 10 pupae sampled at D5, four were positive. Out of 79 adults emerged from D12 to D26, two were positive for MASV at D14 and one at D16. Data are summarized in Table S2.

### 3.3. P. perniciosus Females Infected by Bloodmeal

*Phlebotomus perniciosus* females were infected by a mixture of MASV and blood. The blood meal remains were defecated on day four. Therefore, it was possible to distinguish between non-defecated females (D0) and defecated ones (D5, D7, and D9). As expected, ten non-defecated females D0 had a high rate of infection (90%). In defecated females, the infection rate was lower (18.5%); the MASV was detected in five females out of 27 on D5, D7, and D9 (positivity 20% (2/10), 20% (2/10), and 14%, respectively (1/7)). Data are summarized in Table S3.

### 3.4. P. perniciosus Males and Females Infected by Sugar Meal

**Figure 2:**
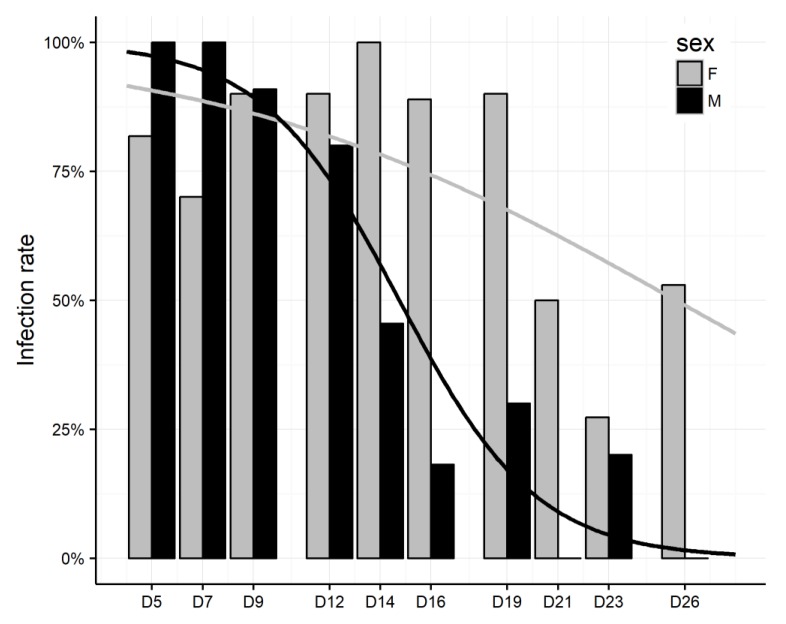
Infection of female and male *Phlebotomus perniciosus* by Massilia virus (MASV) using sugar-meal. Fitted lines for each sex are calculated from the original data according model with quasi-binomial distribution. F = female, M = male.

Of 235 adults *P. perniciosus*, 146 (62%) were positive for MASV RNA. Despite females were more frequently MASV RNA positive than males (71.8% [79/110] vs. 51.5% [51/99]), the difference was not significant. On the other hand, there was signficant difference in progress of infection between females and males (*p* = 0.02). In females, infection rates increased from 80% on D5 up to 100% on D14. From D16 until D26 infection rates were slowly decreasing, from 89% to 52%, respectively, with pronounced drop-down on D23 with an infection rate of 27% (Figure 2). In males, the infection rates reached 100% on D5 and D7. From D9 until D26 infection rates dropped from 91% to 0%, respectively (Figure 2). 

### 3.5. Co-Feeding Experiments

As shown in Table 1, in the CoF1 experiments males and females previously infected via MASV-containing sugar meal on two successive occasions (D0 and D2) have the capacity to transmit MASV orally into the un-infected sugar drop, thus resulting in accumulating contamination of the sugar drop. All drops tested from D4 to D7 were positive for MASV RNA, then contamination continued up to D21 at a lower frequency. Because positivity was observed almost systematically up to D11 (groups 1 and 2), the experiment was continued up to D21 for group 3.

CoF2 was a follow-up of the CoF1 experiment to study if the virus-contaminated sugar drop was capable of infecting naïve sand flies by sugar feeding. Sugar drops exposed to MASV-infected *P. perniciosus* (cage A) were transported into cage B containing uninfected *P. perniciosus* (Table 2). Six sugar drops, collected on D7–9 and D12–14, were accumulated into cage B. Cage B *P. perniciosus* were collected for testing on three occasions: D13 (*n* = 30), D16 (*n* = 30), and D21 (all remaining live sand flies were tested). The number of naïve females and males that became infected by sugar-co-feeding from D13 to D21 ranged from 23% (7/30) to 3.5% (7/200) and from 30% (9/30) to 0% (0/92), respectively.

### 3.6. Susceptibility of Various Sand Fly Species to MASV

**Figure 3:**
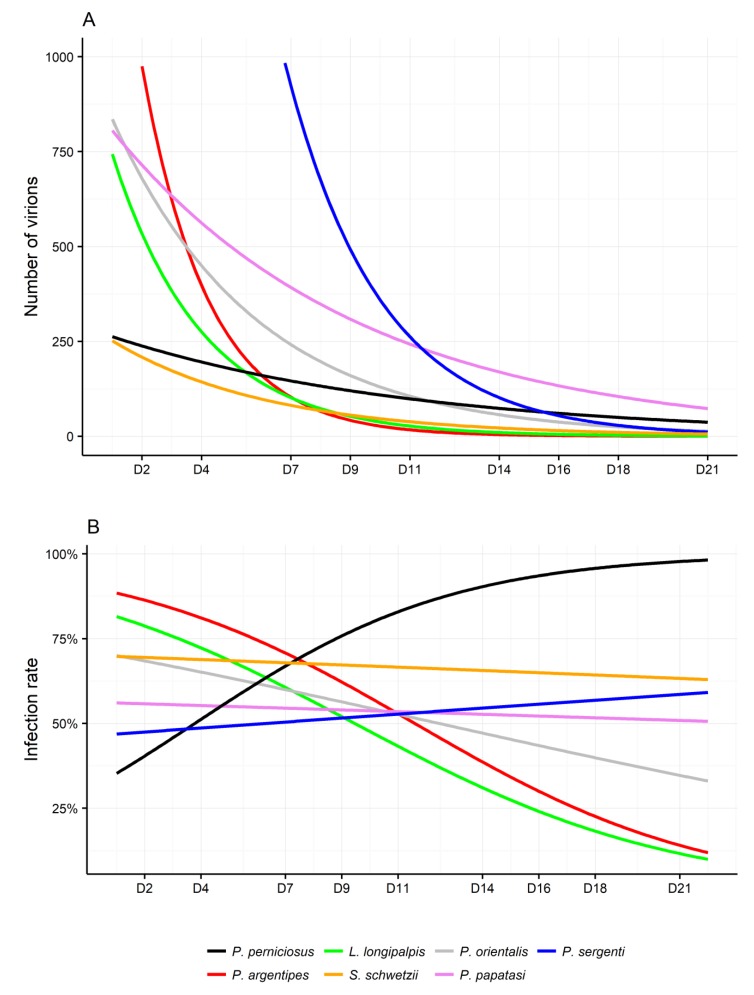
Fitted lines for (**A**) infection rate and (**B**) viral loads in phlebotomine females from different colonies. Fitted lines are calculated from the original data (Table S4) according to models with (**A**) quasi-poisson and (**B**) quasi-binomial distribution. *P.*, *Phlebotomus; L., Lutzomyia; S., Sergentomyia.*

**Figure 4:**
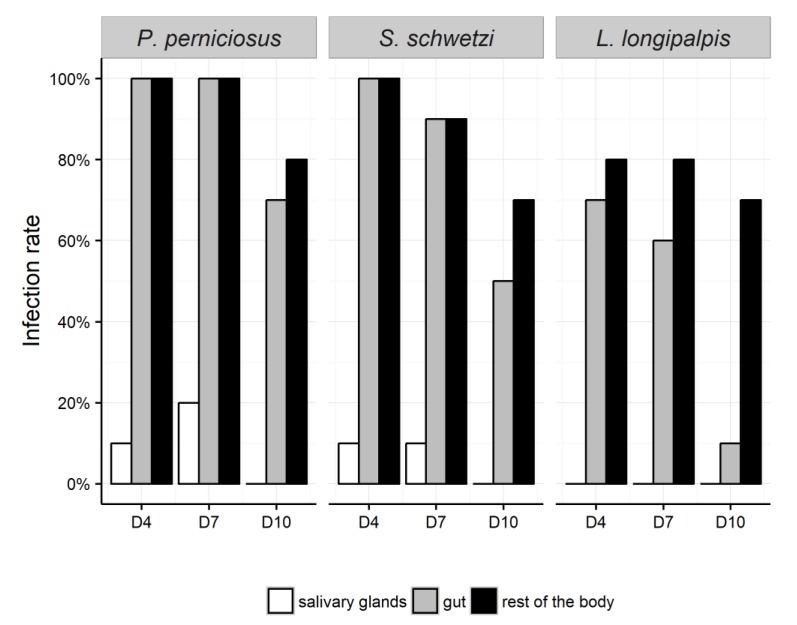
Tissue specifity and infection rate in *Phlebotomus. perniciosus, Sergentomyia schwetzi*, and *Lutzomyia longipalpis*.

The susceptibility to MASV was compared in *P. perniciosus* and six other sand fly species belonging to three genera. Results are summarized in Figure 3 and Figure 4 and in the supplementary data (Figure S1, Tables S4 and S5). The experiment was repeated twice for each species and for each sex and the results were merged for the analysis. MASV was found in both sexes of the seven tested species but infection rate and viral loads differ between species. Also, longevity of various sand fly species kept in laboratory conditions differ. Males have a shorter life-span than females, therefore there is a lack of data for males of some species at later time intervals (*P. orientalis, P. argentipes,* and *S. schwetzi*).

The differences in viral loads and infection rates between the species were analyzed separately for females and males. *Phlebotomus perniciosus* was used as the reference due to the fact that MASV had always been isolated from *P. perniciosus*, which suggests that it is the natural host/vector. 

There were no significant differences in viral loads and progress of infection intensity between males of *P. perniciosus* compared with those of other species. However, for females, the significant differences in viral loads were achieved for *P. sergenti* and *P. argentipes* (Figure 3A). *Phlebotomus sergenti* displayed higher viral loads than *P. perniciosus* females (*p* < 0.001), however, the viral load decreased more rapidly compared with *P. perniciosus* (*p* = 0.02). A similar situation was observed with *P. argentipes* females; higher virus loads (*p* = 0.03), but faster decrease of viral load over time (*p* = 0.05) when compared to *P. perniciosus*. In males, there were no significant differences in infection rates between the seven species. In females, most species tested showed a higher infection rate at the beginning of the experiment when compared to *P. perniciosus*. This difference was significant for *P. argentipes* (*p* = 0.02) and *L. longipalpis* (*p* = 0.03) (Figure 3B). Importantly, all colonies showed significant gradual decrease in infection rate during the experiment when compared to *P. perniciosus* (*P. argentipes p* = 0.004, *L. longipalpis p* = 0.002, *P. orientalis p* = 0.01, *P. papatasi p* = 0.03, *S. schwetzi p* = 0.04). Marginally significant decrease was observed only for *P. sergenti* (*p* = 0.08) (Figure 3B). Importantly, *P. perniciosus* was the only species in which the infection rate grew steadily for the whole time of experiment duration, which suggests transmission between sand flies in the same cage during the experiment.

### 3.7. Tissue Specificity of MASV

Females of three sand fly species were dissected 4, 7, and 10 days after sugar meal infection. The virus was detected and quantified in the salivary glands, the midgut, and the rest of body (Figure 4). Tissue specificity of MASV was analyzed separately for each organ between females of *P. perniciosus* as the “reference colony” and *S. schwetzi* and *L. longipalpis*.

MASV RNA was detected in the salivary glands of *P. perniciosus* and *S. schwetzi* only; 10–20% of females (*n* = 10) dissected on D4 and D7 were positive and there was no significant difference in the viral load. In *L. longipalpis*, no positive salivary glands were found throughout the experiment.

On D4, 100% of *P. perniciosus* and *S. schwetzi* females contained MASV RNA in the gut and in the rest of body. In *L. longipalpis* females, the infection rate on D4 was 75% and 80% in the gut and the rest of the body, respectively (Figure 4). On D10, 70–80% of the female sand flies were positive for MASV RNA in the rest of the body, regardless of the species. In the guts, MASV RNA was detected in 75%, 50%, and 10% of *P. perniciosus, S. schwetzi,* and *L. longipalpis* females, respectively (Figure 4). 

Intensity of infection in the guts was lower for *L. longipalpis* and *S. schwetzi* than in *P. perniciosus*, however, these differences were marginally significant (both *p* = 0.07). In the rest of the body, significantly lower viron loads were found for *S. schwetzi* (*p* = 0.03) and marginally for *L. longipalpis* (*p* = 0.07) when compared to *P. perniciosus*. There was also a marginally significant difference in progress of the infection in the rest of the body between *P. perniciosus* and *S. schwetzi*. The amount of virions decreased slower in *S. schwetzi* than in *P. perniciosus* females (*p* = 0.07) (Table S6). 

## 4. Discussion

Although phleboviruses are transmitted by sand flies in an extensive geographic area, there is limited knowledge about maintenance cycles of the viruses in nature. Vertebrate reservoirs have not been found, but this must be tempered by the fact that few studies addressing this point have been conducted; therefore, the lack of identification of a vertebrate reservoir host does not imply that vertebrate reservoir host(s) does not exist. Recent studies proposed dogs as possible reservoirs [23], but several questions are not answered and the debate remains open. High prevalence rates observed in domestic vertebrates do not imply that they play an epidemiological role in the natural virus cycle. It is likely that, as humans do, they produce antibodies after infection without demonstrating a viremia suitable for transmission to naïve sand flies [7,24]. Early studies have shown that phleboviruses can be transmitted transovarially [16,17,18] and sexually [17] between sand flies. Whether these mechanisms are sufficient in nature for virus perpetuation is uncertain.

The main aim of this study was to investigate the transmission routes of phleboviruses, by using Massilia virus (MASV). MASV belongs to the *Sandfly fever Naples phlebovirus* species that currently includes 13 viruses, among which sandfly fever Naples and Toscana viruses are causing mild to severe illness in infected patients. MASV is a Biosafety Level 2 (BSL2) virus, but its pathogenicity for humans has not been demonstrated and, if anything, appears more limited than that of TOSV. Moreover, MASV is present in a wide geographic area of southwestern Europe. Thus, MASV is a good candidate to be used as a surrogate of TOSV and other pathogenic sandfly borne phleboviruses [14].

Pathways of infection and the developmental stage of the insect can significantly affect tissue tropism of virus, immune response, and outcome of infection [25,26,27]. For arbovirus replication in the vector, the following steps are necessary: (i) Infection of the midgut and infection spread through its epithelium, (ii) dissemination of virus particles to secondary tissues and replication, and (iii) infection of the salivary glands and release of the virus into saliva [28,29]. 

In our experiments, transstadial transmission of MASV from larvae infected at first or fourth instar through larval food was not efficient. In nature, sand fly larvae feed on organic matter [1], thus, they develop an efficient system for control and eventually elimination of bacteria, fungi, and other pathogens from the gut. For example, Sudeep et al. [30] failed to obtain transstadial transmission by feeding second instar larvae of *Culex quinqefasciatus* by fragmented larvae infected with West Nile virus. On the other hand, vertical transmission from mosquitoes and female sand flies to the next generation was repeatedly proven and the virus was detected in the larvae during the whole development until adults [16,31,32,33,34]. Tesh and Modi [16] demonstrated that TOSV was transmitted in *P. perniciosus* during 13 successive generations; however, infection was established from oocytes, not by oral infection of larvae, where the virus has to overcome a number of barriers. 

In Diptera, the majority of extracellular bacteria present in the gut of larvae are not able to survive metamorphosis and only some of them are passed transtadially from larvae to adults [35,36]. Diptera larvae defecate midgut content shortly before pupation and there is massive restructuralization of gut during pupation, including disintegration of larval midgut epithelial cells [37,38]. This process results in massive clearance midgut from bacteria (and possibly also from viruses). In some cases, *Escherichia coli*-GFP and *Pseudomonas*-GFP survive pupation of *Anopheles* mosquitoes hidden in the Malphigian tubules, which open back to the midgut lumen after eclosion of adults [36,39]. We cannot exclude the possibility that a similar mechanism is used by MASV and this may result in low efficiency of transstadial transmission of MASV. However, infection of larvae by the virus through larval food was not efficient and if this route of infection occurs in nature, it is probably very rare or a supplementary phenomenon, which does not participate significantly on maintenance of MASV, and most likely other phleboviruses transmitted by sand flies, in nature. 

In contrast, more promising results were obtained by infecting adult sand flies: (i) Females by blood meal and (ii) both genders by sugar meal. Most of females infected through MASV blood-feeding cleared the virus during blood defecation, thus resulting in a very low percentage of post-defecation infected females. This phenomen of virus elimination may be associated with the peritrophic matrix (PM), which protects the midgut against mechanical damage, pathogens, and toxins. The PM is acellular structure mainly formed by chitin, glycoproteins, and proteoglycans with pores of different size, often ranging around 7–8 nm [40,41,42]. The size of MASV particles is around 80–120 nm [14] so the virus cannot pass through the intact PM. In sand flies, the PM is formed within 6–12 hours after post-blood meal (PBM) and is fully developed after 24 h [43]. Only viral particles located close to midgut epithelial cells after blood feeding and before formation of the PM can infect these cells through microvilli [29]. 

In addition, the elimination of MASV from the gut could be affected by natural gut microflora, either indirectly though stimulation of immune system or directly by microfloral metabolites. In *Aedes aegypti,* after elimination of gut microbiome by antibiotics, Dengue virus titres were twice as high than in non-treated mosquitoes and aseptic individuals showed lower expression of some immune genes like defensin, cecropin, attacin, and gambicin. These results suggest that natural gut microflora stimulate the Toll pathway on a basal level and also exhibit antiviral activity [44,45]. *Chromobacterium* naturally occurring in *Ae. aegypti* decreased susceptibility of mosquitoes to Dengue virus and exhibit anti-viral activities in vitro [46]. 

Interestingly, MASV infection of females and males by sugar meal was significantly more efficient than by blood meal; this is the reason why the following experiments were conducted using sugar-meal infection. Moreover, this way of infection allows the studying of virus infection in both females and males. As far as we are aware, this is the first description of arboviral infection of a dipteran vector using sugar meal. Tang and Ward [47] showed that, at the beginning of sugar-feeding of *L. longipalpis, a* small amount of sugar was passed directly to the thoracic midgut which led to closure of the stomodeal valve and the rest of sugar was deflected to crop. This means that infection is initiated either from this “first drop” or from the sugar meal temporarily stored in the crop. 

Males and females that are infected with MASV repeatedly spit MASV into sugar solution during feeding from D4 until D21. Similar results were observed also for viruses transmitted by *Culex annulirostris* and *Culex gelidus* [48]. Interestingly, despite MASV infection being found in *P. perniciosus* salivary glands until D7 post infection, virus particles were expectorated into sugar solution until D21. Moreover, virus regurgitated into sugar meal by sand flies was efficiently transmitted to naïve females and males. These findings suggest that MASV is not released only with saliva but also by regurgitation from the alimentary canal. Unpublished data (Charrel, personal communication) demonstrate that MASV survives for at least 24 h in wet conditions, like plant nectar. Almost 30% of *P. perniciosus* (both males and females) get MASV from the sugar with expectorated virus. The same mechanism should operate with plant sap in nature, where phleboviruses can be transmitted from infected to uninfected sand flies during plant sugar feeding either simultaneously (as in non-viremic co-feeding shown with ticks and tick-borne encephalitis virus) or successively; this may represent an important part of the natural cycle of phleboviruses. This suggests that horizontal transmission of phleboviruses is possible when infected sand flies are feeding on sugar sap; it is important to underline that sand fly-feeding on sugar solutions of plant origin or honey dew is frequent for both sexes [49,50], while only females take a blood meal for egg maturation. Demonstration that males can get infected from a virus-containing sugar source and that these newly-infected males are capable to contaminate previously uninfected sugar sources suggest that horizontal transmission of viruses is a pathway that should be considered in nature and that females can get infected through this mechanism. Further studies are needed to explore how important this mechanism can be in the natural cycle, but our results show that this deserves to be investigated. In addition, males that get infected from sugar sources may also transmit the virus to females via venereal transmission, which also needs to be explored further. A weakness of this study is the fact that, although detection of MASV RNA is not disputable, infectiousness has not been addressed because of university regulations and biosafety reasons. In light of these promising results, this has to be considered.

Very little is known about the specificity of phleboviruses towards different sand fly species. We tested MASV infection in seven species belonging to three genera, *Lutzomyia, Phlebotomus,* and *Sergentomyia*. In males, no significant differences were found in viral loads and infection rates between various sand fly species. In contrast, in females, viral loads and infection rates differed between species. Unlike other sand fly species, the infection rate in *P. perniciosus* grew steadily until D15 and then remained stable until the end of the experiment. The fact that the infection rate increases even after the females were denied feeding on infected sugar (given only at D0 and D2) could be explained by infection through co-feeding, as previously described. Our results indicate that MASV survived in all tested species of sand flies for weeks. Hovewer results with *L. longipalpis* suggest that the virus can not replicate in the New World sand flies. As TOSV has been isolated from other species belonging to the *Larroussius subgenus*, such as *P. perfiliewi* and *P. neglectus*, future studies should take these aspects into consideration and determine whether virus replication occurs. The fact that species other that *P. perniciosus* can also be infected suggest that *P. sergenti* for instance is likely to play a role in the transmission in nature. These results are in agreement with recent studies which detected Toscana virus RNA not only in *P. perniciosis* and *P. perfiliewi*, but also in *P. sergenti* (in Morocco [13]) and *P. neglectus* (in the Balkans [12]).

Interestingly, our results are congruent to point to *P. perniciosus* as the species in which replication of MASV is optimal, according to the criteria which were studied: (i) Rates of MASV infection is largely above 50% in males and females exposed to virus-infected sugar meal; (ii) this infection rate is consistent for 12 days during which transmission is possible; (iii) infected sand flies regurgitate MASV into sugar meal upon feeding, from which naïve *P. perniciosus* get infected; (iv) in *P. perniciosus*, infection rate grew steadily throughout the experiment, suggesting active transmission between sand flies of the same cage.

In conclusion, we showed that MASV is not transmitted efficiently between different *P. perniciosus* development stadia or through blood meal in *P. perniciosus* adults. The latter relies on a vertebrate species in which viremia is high enough to infect naïve sand flies at a high frequency. By showing that blood meal transmission does not result in a high proportion of infected females, our results indirectly support the theory that vertebrates represent dead-end hosts without an important role in the virus life cycle [15]. Transmission through blood feeding between vector and vertebrates can be a supplementary way of circulation of MASV, and also for other phleboviruses transmitted by sand flies, but different mechanisms other than insect–vertebrate cycles have to be hypothesized. The most efficient route of infection was observed during oral feeding on infected sugar. Infected sand flies can regurgitate virus particles into the source of sugar during feeding (plant sap in nature) which subsequently becomes a source of the virus that can be exposed to naïve sand flies, regardless of their gender. Results suggest that non-arboviral transmission can play a significant role in the maintenance of MASV and most likely other sand fly-borne phleboviruses. Further studies are merited to investigate further to what extent this type of transmission can sustain virus maintenance in nature. 

## Figures and Tables

**Table 1:** Detection of MASV in sugar drops exposed to sand flies fed on MASV infected sugar meals at D0 and D2. Sugar drops were tested from D4 to D11 (groups 1 and 2), and D4 to D21 (group 3). +, MASV RNA positive; −, absence of MASV RNA.

Group	D4	D5	D6	D7	D10	D11	D12	D13	D14	D17	D18	D19	D20	D21
Females 1	+	+	+	+	−	−								
Females 2	+	+	+	+	−	+								
Females 3	+	+	+	+	+	+	−	+	+	−	−	+	+	−
Males 1	+	+	+	+	−	+								
Males 2	+	+	+	+	−	+								
Males 3	+	+	+	+	+	+	−	+	−	−	−	+	−	+

**Table 2:** Infection rate of females/males infected by “co-feeding on sugar”. Infection rate of females/males *P. perniciosus* infected by sugar meal transported from cage with infected females/males (A) by MASV into cage with non-infected females/males (B). neg, negative; F = female; M = male.

Cage B	D7	D8	D9	D12	D13	D14	D15	D16	D21
F cage: RNA copies in sugar drop	440	neg	192	199	neg	3			
Infected F/tested F					7/30 (23%)			5/30 (17%)	7/200 (3.5%)
M cage: RNA copies in sugar drop	1919	neg	2258	142	1	neg			
Infected M/tested M					9/30 (30%)			2/30 (7%)	0/92 (0%)

## Supplementary Materials

The following are available online at https://www.mdpi.com/1999-4915/11/4/332/s1, Figure S1: Susceptibility of females (grey bars) and males (black bars) of seven sand fly species to Massilia virus (MASV), Table S1: *Phlebotomus perniciosus* L1 infected with larval food (2 doses at D0 and D2), Table S2: *Phlebotomus perniciosus* L4 infected with larval food (one dose at D0), Table S3: *P. perniciosus* females infected by bloodmeal (one dose at D0), Table S4: Susceptibility of females of *P. perniciosus*, *P. orientalis*, *P. papatasi*, *P. sergenti*, *P. argentipes*, *S. schwetzi* and *L. longipalpis* to MASV, Table S5: Susceptibility of males of *P. perniciosus*, *P. orientalis*, *P. papatasi*, *P. sergenti*, *P. argentipes*, *S. schwetzi* and *L. longipalpis* to MASV, Table S6: Tissue specificity of MASV in three selected species. Intensity of infection in gut, salivary glands and rest of body in females *P. perniciosus, S. schwetzi* and *L. longipalpis*.
