# Supplementary material for: Experimental Infection of Sand Flies by Massilia Virus and Viral Transmission by Co-Feeding on Sugar Meal
Source: Viruses. 2019 Apr 9;11(4):332. doi: 10.3390/v11040332 (PMC6520868; doi:10.3390/v11040332)
Supplement: Supplementary file 1 [file viruses-11-00332-s001.zip › Figure S1.pdf]

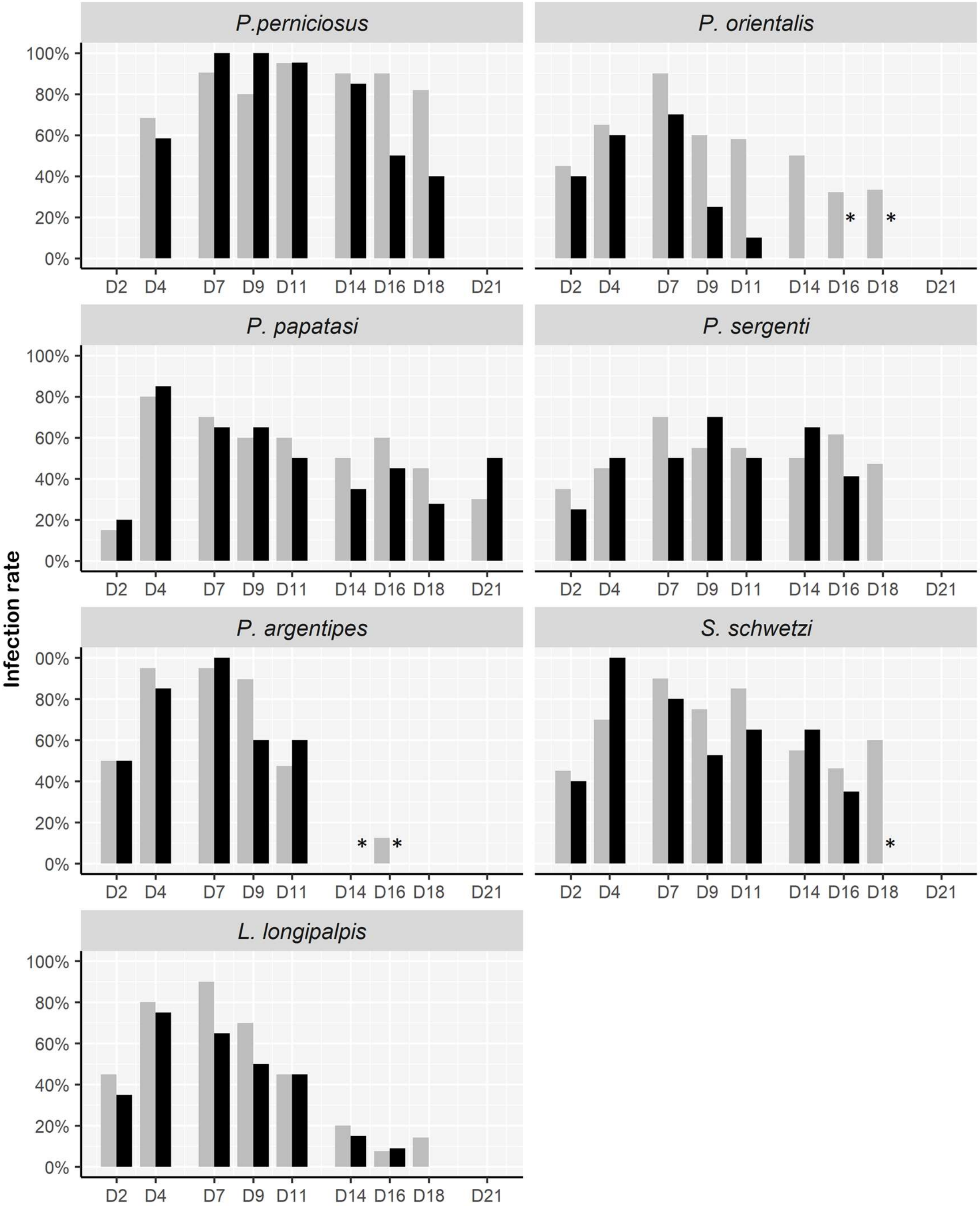

Figure S1. Susceptibility of females (grey bars) and males (black bars) of seven sand fly species to Massilia virus (MASV). * no living males were available by these intervals. *P., Phlebotomus; S., Sergentomyia; L., Lutzomyia.*
