# Supplementary material for: Experimental Infection of Sand Flies by Massilia Virus and Viral Transmission by Co-Feeding on Sugar Meal
Source: Viruses. 2019 Apr 9;11(4):332. doi: 10.3390/v11040332 (PMC6520868; doi:10.3390/v11040332)
Supplement: Supplementary file 1 [file viruses-11-00332-s001.zip › Supplementary Tables.pdf]

S1 Table. ***Phlebotomus perniciosus*** **L1 infected with larval food (2 doses at D0 and D2)**

**Developmental stage**	**Sampling day**	**Nb positive / tested / %**
Pre exposure	D0	0/20
larvae	D2	0/20
larvae	D5 / D3	**6/18/33.3%**
larvae	D12 / D9	0/20
larvae	D19 / D16	0/20
pupae	D26 / D23	**1/20/5%**
emerged adults	D33 / D30	0/17/0
emerged adults	D35 / D32	**1/29/3.4%**
emerged adults	D37 / D34	0/86/0%
emerged adults	D38 / D35	0/234
emerged adults	D40 / D37	0/143
emerged adults	D42 / D39	0/126
emerged adults	D45 / D42	0/102
emerged adults	D47 / D44	0/22
emerged adults	D49 / D46	0/19
emerged adults	D52 / D49	0/11
emerged adults	D54 / D51	0/2
emerged adults	D56 / D53	0/5

S2 Table. ***Phlebotomus perniciosus*** **L4 infected with larval food (one dose at D0)**

**Developmental stage**	**Sampling day**	**Nb positive / tested / %**
larvae	D0	0/20
larvae	D2	0/10
pupae	D5	**4/10/40%**
emerged adults	D12	0/2
emerged adults	D14	**2/11/18.2%**
emerged adults	D16	**1/20/5%**
emerged adults	D19	0/37
emerged adults	D21	0/5
emerged adults	D23	0/3
emerged adults	D26	0/1

S3 Table. ***P. perniciosus*** **females infected by bloodmeal (one dose at D0)**

**Developmental stage**	**Sampling day**	**Nb tested**
adult females pre-exposure to infected blood		0/20
adult females non defecated	D0	**9/10/90%**
adult females defecated	D5	**2/10/20%**
adult females defecated	D7	**2/10/20%**
adult females defecated	D9	**1/7/14.3%**

S4 Table. Susceptibility of females of *P. perniciosus*, *P. orientalis*, *P. papatasi*, *P. sergenti*, *P. argentipes*, *S. schwetzi* and *L. longipalpis* to MASV. neg = negative

		D2	D4	D7	D9	D11	D14	D16	D18	D21
P. perniciosus	infected/total	0/20	15/22	19/21	16/20	19/20	18/20	18/20	18/22	
	% infected	0	68.2	90.5	80	95	90	90	82	
	mean number of RNA copies	**neg**	**149**	**600**	**89**	**105**	**27**	**8**	**5**	
P. orientalis	infected/total	9/20	13/20	18/20	12/20	11/19	10/20	9/28	2/6	
	% infected	45	65	90	60	57.9	50	32.1	33.3	
	mean number of RNA copies	**555**	**600**	**283**	**125**	**51**	**87**	**42**	**13**	
P. papatasi	infected/total	3/20	16/20	14/20	12/20	12/20	10/20	12/20	9/20	3/10
	% infected	15	80	70	60	60	50	60	45	30
	mean number of RNA copies	**1385**	**139**	**134**	**49**	**366**	**75**	**53**	**443**	**57**
P. sergenti	infected/total	7/20	9/20	14/20	11/20	11/20	10/20	8/13	8/17	
	% infected	35	45	70	55	55	50	61.5	47.1	
	mean number of RNA copies	**5714**	**769**	**786**	**874**	**324**	**130**	**38**	**64**	
P. argentipes	infected/total	10/20	19/20	19/20	17/19	9/19	0/8	1/8		
	% infected	50	95	95	89.5	47.4	0	13		
	mean number of RNA copies	**1051**	**277**	**136**	**56**	**16**	**neg**	**5**		
S. schwetzi	infected/total	9/20	14/20	18/20	15/20	17/20	11/20	6/13	6/10	
	% infected	45	70	90	75	85	55	46.2	60	
	mean number of RNA copies	**230**	**95**	**95**	**84**	**35**	**16**	**5**	**15**	
L. longipalpis	infected/total	9/20	16/10	18/20	14/20	9/20	4/20	1/13	1/7	
	% infected	45	80	90	70	45	20	7.7	14.3	
	mean number of RNA copies	**622**	**129**	**179**	**34**	**12**	**24**	**5**	**3**	

S5 Table. Susceptibility of males of *P. perniciosus*, *P. orientalis*, *P. papatasi*, *P. sergenti*, *P. argentipes*, *S. schwetzi* and *L. longipalpis* to MASV. neg = negative

		D2	D4	D7	D9	D11	D14	D16	D18	D21
P. perniciosus	infected/total	0/10	14/24	21/21	20/20	20/21	17/20	10/20	8/20	
	% infected	0	60	100	100	95	85	50	40	
	mean number of RNA copies	**neg**	**94**	**418**	**153**	**82**	**29**	**2**	**5**	
P. orientalis	infected/total	8/20	12/20	14/20	5/20	2/20	0/10			
	% infected	40	60	70	25	10	0			
	mean number of RNA copies	**103**	**106**	**112**	**13**	**20**	**neg**			
P. papatasi	infected/total	4/20	17/20	13/20	13/20	10/20	7/20	9/20	5/18	5/10
	% infected	20	85	65	60	50	35	45	27.8	50
	mean number of RNA copies	**162**	**525**	**271**	**81**	**36**	**37**	**78**	**273**	**430**
P. sergenti	infected/total	5/20	10/20	10/20	14/20	10/20	13/20	7/17	0/1	
	% infected	25	50	50	70	50	65	41.2	0	
	mean number of RNA copies	**302**	**271**	**433**	**160**	**116**	**66**	**86**	**neg**	
P. argentipes	infected/total	10/20	17/20	20/20	9/15	3/5				
	% infected	50	85	100	60	60				
	mean number of RNA copies	**258**	**110**	**110**	**43**	**26**				
S. schwetzi	infected/total	8/20	20/20	16/20	10/19	13/20	13/20	7/20		
	% infected	40	100	80	52.6	65	65	35		
	mean number of RNA copies	**134**	**56**	**28**	**12**	**36**	**14**	**11**		
L. longipalpis	infected/total	7/20	15/20	13/20	10/20	9/20	3/20	1/11	0/10	
	% infected	35	75	65	50	45	15	9.1	0	
	mean number of RNA copies	**320**	**135**	**38**	**12**	**114**	**46**	**4**	**neg**	

S6 Table. Tissue specificity of MASV in three selected species. Intensity of infection in gut, salivary glands and rest of body in females *P. perniciosus*, *S. schwetzi* and *L. longipalpis*.

	**Viral load**	**D4**	**D7**	**D10**
P. perniciosus	salivary glands	12	12	neg
	midgut	1007	48	17
	rest of body	6354	496	227
S. schwetzi	salivary glands	11	7	neg
	midgut	262	30	32
	rest of body	1081	345	412
L. longipalpis	salivary glands	neg	neg	neg
	midgut	176	51	11
	rest of body	1202	506	71
